# Design of Proactive Interaction for In-Vehicle Robots Based on Transparency

**DOI:** 10.3390/s22103875

**Published:** 2022-05-20

**Authors:** Jianmin Wang, Tianyang Yue, Yujia Liu, Yuxi Wang, Chengji Wang, Fei Yan, Fang You

**Affiliations:** 1 Car Interaction Design Lab, College of Arts and Media, Tongji University, Shanghai 201804, China; wangjianmin@tongji.edu.cn (J.W.); michaelyue0812@tongji.edu.cn (T.Y.); liuyujia@tongji.edu.cn (Y.L.); wangyuxi@tongji.edu.cn (Y.W.); laoji@tongji.edu.cn (C.W.); 2 Shenzhen Research Institute, Sun Yat-Sen University, Shenzhen 518057, China; 3 Nanchang Research Institute, Sun Yat-Sen University, Nanchang 330224, China; 4 Ulm University, 89081 Ulm, Baden-Württemberg, Germany; fei.yan@uni-ulm.de

**Keywords:** interaction design, transparency, proactivity, in-vehicle robots

## Abstract

Based on the transparency theory, this study investigates the appropriate amount of transparency information expressed by the in-vehicle robot under two channels of voice and visual in a proactive interaction scenario. The experiments are to test and evaluate different transparency levels and combinations of information in different channels of the in-vehicle robot, based on a driving simulator to collect subjective and objective data, which focuses on users’ safety, usability, trust, and emotion dimensions under driving conditions. The results show that appropriate transparency expression is able to improve drivers’ driving control and subjective evaluation and that drivers need a different amount of transparency information in different types of tasks.

## 1. Introduction

With the rapid development of intelligent vehicles, drivers’ requirements of more intelligent assistances from the cockpit have increased. More vehicles are equipped with virtual image voice assistants or vehicle robots with a physical entity, etc. These in-vehicle intelligent assistants enhance the intelligence level of the cockpit and can execute diverse tasks. The interaction between human and in-vehicle robots is considered as an integration of a complex social and technical system [1], which needs an advanced model to improve safety and trust in autonomous vehicles [2].

Anthropomorphism and proactivity have been widely studied for the future in-vehicle robots. A study by Waytz et al. [3] showed that a more anthropomorphic cockpit can increase human trust and is perceived to have more human-like mental abilities. It also showed that an anthropomorphic robot’s voice response can increase trust, pleasure, and dominance of the situation compared to mechanical voice response [4]. However, there are still concerns about communication barriers for such robots. The accuracy and validity of the output produced by intelligent systems can be problematic because it is difficult for the operator to interpret the output [5]. Part of the reason is that humans have limits to understand the proactivity of robots [6]. The study showed that people are more receptive to the support provided by robots with moderate proactivity than those with high or low proactivity [7]. Another reason is that the robot interaction design does not match human cognition.

An important condition for a robot to be able to interact fluently with humans is that the two can share a common cognitive framework [8] and form a coherent mental expectation during the interaction without adding additional learning costs. Therefore, in order to promote a common mental model between human operators and automated systems, in-vehicle robots should be designed according to human cognition to improve communication efficiency and trust. Human cognitive architectures have also been increasingly applied to the intelligent architecture of robots in recent years [9]. Some studies have highlighted system transparency as a potential barrier to trust and technology acceptance [10]. Lee and See [11] also suggested that autonomous systems should communicate their capabilities and limitations as much as possible in order for humans to develop appropriate trust and dependence. It is important to make the state of the autonomous system transparent to humans under conditions consistent with human cognition. Chen and colleagues [12] proposed the situation awareness-based agent transparency theory (SAT) to help establish a common mental model between human and machine, which further generates assistance to the human decision-making process and enhances task performance.

Using cognitive theory to design in-vehicle robots to provide appropriate expression to humans has not been widely studied. This paper applies situation awareness-based agent transparency theory to design in-vehicle robots. The objective of this paper is to explore the appropriate amount of transparency information that can assist human decision making by conducting experiments. Because the inappropriate amount of information while driving, especially in critical situations can affect safety, usability, workload, and trust, the following hypotheses are made.

1. The expressed in-vehicle robot’s information to drivers needs to be selective.
2. The appropriate amount of transparency information for different driving situations needs to be determined.

In order to test these hypotheses, in-vehicle robot transparency design has been made at three levels of perception, comprehension and projection based on SAT theory, including channels of voice and visual in the design. Then, experimental evaluation of the transparency design hypothesis has been carried out in selected driving scenarios. After analyzing the experimental results, conclusions of transparency design of in-vehicle robot proactive interaction in different scenarios have been made.

The contribution of this paper is that it gives results on how an appropriate level of transparency expression affects drivers’ driving control and subjective measurement. It also identifies that drivers need a different amount of transparency information in different types of scenarios. Results can provide guidance to help design in-vehicle robot interface with appropriate transparency.

## 2. Related Work

As human–robot interaction (HRI) becomes more complex, many guidelines and design criteria have been developed depending on the specific applied scenarios [13]. A shared mental model can lead to higher levels of performance [14]; hence, effective team member communication needs to be considered in the design process of proactive robot interaction. Transparency plays an important role in building an understanding of human–robot interaction and can contribute to the interaction design of in-vehicle robots.

### 2.1. Proactive Interaction

As intelligent systems become complex, researchers have begun to focus on proactivity. There is some consensus in the research field regarding the definition of proactivity, mostly in terms of the role and state of the initiator of the interaction. Keith et al. [15] argued that a proactive system should be a representative of the user and able to initiate behavior without user commands. In proactive interaction, human operators can achieve more supervisory control rather than active control [16]. However, the change in control mode does not mean that the human’s job becomes easier. Highly proactive robots also have a greater influence on human decision making [17]. More cognitive factors need to be considered in the design. The appropriateness of the quality and quantity of information transfer have to be considered.

Multimodal interaction is also widely used in proactive interaction scenarios. For an anthropomorphic robot, dual-channel interaction between speech and vision is crucial [18] and can significantly affect the mutual understanding between human and robot. In the context of Industry 4.0, the development of multimodality in the driving environment is also reflected in the research of many different devices, including VR, AR, and robotics [19]. Experiments by Williams et al. [20] also demonstrated that multimodal interaction can be more effective than separate channel interaction in reducing driving workload and distractions as well as in enhancing emotional experience. The importance of multimodal interaction for proactive interaction has also been shown in previous studies [21].

The core of contemporary research on proactive interaction focuses on the technology domain. Most of them are based on the decision making of artificial intelligence models to enhance situation awareness, consciousness perception, and emotion perception. Specifically, in the in-vehicle scenario, proactive interaction can help users collect and process information in the environment, therefore reducing the user’s information processing burden. A further study [22] proposed human–autonomous team cooperation based on robot initiative to monitor and receive feedback from each other.

Proactive interaction is of high importance due to the development of intelligent interface. However, the current work on proactive interaction is focused on the related HRI system in the technical field [23], which is dedicated to finding solutions from the perspective of AI. The explorations of proactive interaction design from the perspective of cognitive theory are sparse.

### 2.2. Transparency

Understanding the reasoning process behind the output of an intelligent system in a dynamic environment is of great importance [8]. Van Dongen et al. [24] found that the perceptibility process of participants to the reasoning process of a decision aid system has a significant impact on their reliance on its recommendations.

In the context of automation, an understanding of the behavior of technical agents is important to ensure good interactions between human and technical agents. This understanding is often referred to as “transparency”. Lyons [14] argues that transparency can facilitate optimal calibration between humans and autonomous systems. The design of the appropriate amount of information on different display devices in the driving environment has also been explored [25].

In terms of information content, clearer and more accurate information delivery can enhance human trust [26]. It has been mentioned [27] that the information provided by the proactive party, the machine, should be highly transparent to the user to allow the user to identify and understand it quickly. Russell et al. [28] mentioned that the characteristics of autonomy that intelligent agents should possess include observation of the environment, action on the environment, and guidance of the activity. Lee [29] suggested that in order to increase the transparency of automation to the operator, system designers should make the 3Ps (purpose, process, and performance) of the system and its history visible to the operator.

Endsley’s situation awareness (SA) theory [30] proposes three levels including SA Level 1, perception of elements in the environment, SA Level 2, comprehension of these elements, and SA Level 3, projection of their state in the near future. Based on SA theory, Chen et al. proposed the situation awareness-based agent transparency theory (SAT) [12] for explaining what information contributes to transparency. The SAT model argues that as the agent is involved in the execution of the human task, the human needs to be situationally aware of the agent and the environment, which can be achieved through the agent’s transparency. Situation awareness-based agent transparency theory defines agent transparency as a descriptive quality of an interface, where the operator understands the intention, reasoning process, and future plans of an intelligent agent.

The SAT model also consists of three levels. At the SAT Level 1, the perception level, the operator should be provided with the agent’s goals and its perception of the environmental situation. At the SAT level 2, the comprehension level, the operator should be provided with the agent’s understanding of the situation and the reasoning process of the action. At the SAT level 3, the projection level, the operator should be provided with the agent’s projection of the future outcome. All three levels of the SAT model describe the information that the agent needs to convey to maintain transparent interactions with humans. The operator is, therefore, able to understand the agent’s intentions, reasoning process, and predicted outcomes, which leads to better information sharing and a common mental model of their communication. 

The presence or absence of each level of information in the SAT determines whether the user is able to understand the perception, comprehension and projection of the current information expressed by robots. The design needs to consider that each level of information can be combined to obtain an appropriate transparency that promotes mutual human–robot understanding without increasing the workload. Conducting phase analysis through an SAT model is a way to contribute to transparency between humans and robots. The usability of the intelligent system, the emotional experience of humans, and the level of trust can be measured. Related studies have also applied the SAT model for interface design to conduct explorations, including robot action interfaces and UxV action interfaces [31,32].

## 3. Transparency Design of Proactive Interaction

According to the theory of situation awareness, the human cognitive process includes three levels: perception, comprehension, and projection [30]. In the human–robot interaction process, the necessity of transparency in the three levels is reflected in the human in robot. At the same time, anthropomorphism and proactivity require robots to present multiple channels such as voice and visual.

**Figure 1:**
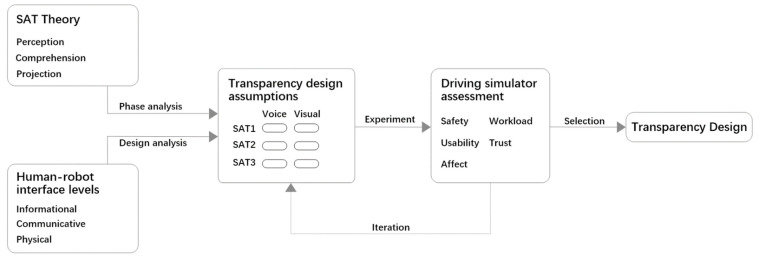
Research framework.

In the design of in-vehicle human–robot interaction, it is necessary to consider the appropriateness of the amount of information in different channels and different SAT levels. The impact on several aspects needs to be examined such as driving safety, usability, and emotion. The research framework is shown in Figure 1. We apply SAT theory to conduct analysis to determine which SAT level the information is in. Combined with human–robot interface levels [14], the transparency design assumptions for the in-vehicle robot were carried out based on the proactive interaction scenario of the in-vehicle robot. The design assumptions are then evaluated through experiments, using a driving simulator.

### 3.1. Human–Robot Interface Levels

In order to promote appropriate transparency, Lyons argues that an opportunity to foster transparency between the human and the robot is from the human–robot interface. The human–robot interface includes three levels: informational, communicative, and physical [14]. Each level covers a portion of what transparency design needs to consider. The levels in SAT theory are distinct from the levels of the human–robot interface. The SAT levels focus on the interaction process, where the human–computer interaction process can be well divided into three stages. Meanwhile, human–robot interface levels function as a guidance on the specific design to determine the existence and the amount of specific information.

For specific designs, interface features at the informational level need to be considered to avoid too much information or non-intuitive displays, which may confuse and frustrate the user of the robotic system. Interface features at the communication level need to be considered to avoid the robot’s inappropriate responses, which affects user trust and performance. Interface features at the physical level may include the robot’s emotional expression, effectively describing the robot’s emotional state.

In transparency design assumptions, the amount of information and information intuitiveness should be considered at the informational level. At the communication level, communication smoothness and response timeliness should be considered. At the physical level, emotional expressions should be considered.

### 3.2. Transparency Design Assumptions

Human–robot transparency design requires identifying certain interaction timing. The identification enables the design of in-vehicle robots to present the driver with the appropriate level of transparency to facilitate the driver’s understanding. The in-vehicle robots give appropriate information that conforms with driver’s cognition, thus resulting in transparency of the entire interaction process. We have explored the possible design combination and excluded the designs that were clearly unreasonable for each interaction timing in a given scenario. For example, considering communication smoothness and response timeliness, the information can be neither too much or too little. Then, the design pattern for each transparency level emerged, forming the transparency design assumption.

Transparency was first analyzed from the perspective of information intuitiveness. From design exploration we conducted before the experiment, we found that when users were given information at the comprehension and perception levels, they can accurately identify the existence of the projection level. However, when the information at the projection level existed alone, users defaulted to the existence of perception and comprehension levels of information. It showed that subjects were unable to cognitively recognize low-level transparency information in the presence of high-level transparency information. When analyzed at the physical level, emotional expression providing information at the perception and comprehension levels may affect subjects’ emotions. Therefore, perception and comprehension levels of information should still be taken into account in the transparency design assumptions.

In the proactive interaction condition of the in-vehicle robot, it has more information that needs to be shared with people, and therefore, it requires a higher transparency of expression. At the perception level, considering emotional expression from the physical level, voice cues can increase the robot’s anthropomorphism and enhance human emotions in the corresponding scenarios. At the comprehension level, considering the informational level aspect, the robot needs to covey the information it perceived to people, and how it comprehends the information needs to be transparent in the voice channel. At the perception and comprehension levels, information in the visual channel, the information intuitiveness, and task fluency are considered, and the comprehension level information is chosen to be retained. At the projection level, considering information intuitiveness, the voice channel gives easier understanding compared to the visual channel, so that the projection information is designed to be expressed through the voice channel. Therefore, the design of the projection level is assumed to include the voice channel information. It is expected that the visual channel projection level information can play an auxiliary role to the voice channel information to strengthen the robot’s expressiveness. Incorporating the communication smoothness into the comprehensive consideration, the presence or absence of visual channel SAT3 level information can be examined. The design assumptions are listed in Table 1 below, where the question mark represents its inability to analyze the necessity of its existence from the design perspective and the need for experimental verification.

To summarize the above research approach, the transparency design assumes that the human–robot communication can be more understood by each other and interaction can be more efficient. What kind of design pattern can reach the above goal needs to be determined. Our research approach is first to use the SAT model to analyze the stages, splitting the entire human–robot interaction process into three stages. After the stages were analyzed, we adopt the human–robot interface levels of informational, communicative, and physical guidance. We then further expand human–robot interface levels into the amount of information, information intuitiveness, communication smoothness, response timeliness, and emotional expression for each stage to analyze the information under voice and visual channels and SAT levels.

## 4. Experiment

### 4.1. Participants

Thirty subjects (25 males and 5 females), with the age ranging from 22 to 40 years (M=28.2, SD=5.83), were selected for the experiment. All subjects had proficient driving experiences, and 16 of them drove 2–3 days per week and 14 subjects drove 4 days per week and above. Subjects were recruited through an online screening process, and their driving experience ranged from 1 to 10 years (M=5.8, SD=2.9). Of these, 21 subjects had never used an in-vehicle robot before, nine participants had experienced one once before, and there were no existing in-vehicle robot users. Therefore, the participants were all regarded as novice in-vehicle robot users. The independent variable of the experiment was the degree of transparency, and between-group experiments were designed. The age and gender distribution of subjects were adjusted according to the number of experimental groups in the task so that the demographic attributes of the subjects were as balanced as possible.

### 4.2. Design of Experiment

The experiment focuses on the proactive interaction scenarios of in-vehicle robots. After conducting real car research and interviews, we collected the scenarios which were used frequently and more representative. The final experimental tasks for proactive interaction with the in-vehicle robot were identified as telephone and speeding. The telephone task required the driver and the in-vehicle robot to complete a non-driving task together. The speeding task required the driver and the in-vehicle robot to complete a critical task together. The experiment used between-subjects design. The aim of the experiment is to explore the relationship between the change in transparency levels, the information quantity inside each level and the driving behavior. Two tasks with different scenarios also enable a comparative exploration of the optimal information quantity design for different scenarios. In the two tasks, the SAT Level 1 was a perception to speeding or incoming call. The SAT Level 2 was a comprehension of the situation mentioned above. The SAT Level 3 was a projection on the driver’s nearest action, expressing as a suggestion or question.

In the telephone task, the experimenter simulated a phone call from the subject’s friend Wang. Then, the robot took the initiative to alert and ask the person whether to answer it. The specific experimental group design is as follows:

1. Group 1 contained SAT Level 2 and SAT Level 3 messages. The robot told the driver ‘Wang is calling you’, with a pleasant expression. Then, the robot asked the driver ‘Would you like to answer’, with a phoning expression.
2. Group 2 contained SAT Level 1, SAT Level 2 and SAT Level 3 messages. SAT Level 1 voice channel message ‘Ring’ was added.

**Figure 2:**
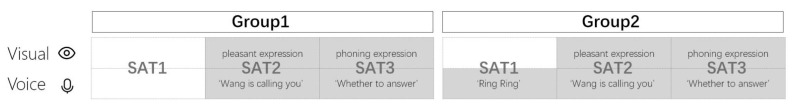
Telephone task experimental group setup. The gray choices represent information that appeared at a certain level in the experimental group, and the white choices represent that there is no information in that level.

The set up for the telephone task is shown in Figure 2. In total, 12 males and 3 females were assigned to experimental group 1; 13 males and 2 females were assigned to experimental group 2.

In the speeding task, the subject was asked to drive in the left lane at a speed of 30 km/h and then accelerated to 70 km/h. Once the speed was above 60 km/h, the robot took the initiative to remind the driver of the speed limit. The specific experimental group design is as follows:

1. Group 1 contained SAT Level 2 and SAT Level 3 messages. The robot told the driver ‘The speed limit ahead is 60 km/h, you have exceeded the speed limit’, with a fear expression. Then, the robot suggested the driver ‘Slow down please’.
2. Group 2 also contained SAT Level 2 and SAT Level 3 messages. Based on having all the information in the experimental group 1, a speed limit expression, containing an SAT Level 3 visual channel message, was added.
3. Group 3 contained SAT Level 1, SAT Level 2, and SAT Level 3 messages. Based on having all the information in the experimental group 1, an SAT Level 1 voice channel message ‘Oops’, which expressed robot perception to the driver, was added.

**Figure 3:**
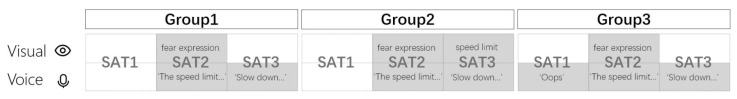
Speeding task experimental group setup. The gray choices represent information that appeared at a certain level in the experimental group, and the white choices represents that there is no information in that level.

The experimental group setup for the speeding task is shown in Figure 3. In total, 8 males and 2 females were assigned to experimental group 1 and experimental group 2; 9 males and 1 female were assigned to experimental group 3.

### 4.3. Measurement

The behavior of the in-vehicle robots will draw the attention of the drivers, even if robots perform decisions that conform with the drivers’ cognition and give them more helpful information. Appropriate design of human–robot interaction strategies based on human cognitive factors can help compensate for human limitations to achieve safety [27]. Therefore, it is necessary to examine the multi-channel transparency design assumptions of in-vehicle robots to ensure driving safety, to improve task execution efficiency, and to enhance drivers’ trust in the robot.

Harbluk et al. [33] showed that the driver’s visual behavior and vehicle control changed when performing tasks with different cognitive requirements. The driver’s visual behavior data and vehicle data were collected while participants were performing tasks. The visual behavior data included the total saccades time (times) and total fixation time (seconds), which were extracted from recorded videos. The vehicle data included vehicle speed (km/h) and driveway offset (dm), which measured subjects’ driving control in the vertical and horizontal directions, respectively. In terms of subjective data, post-task questionnaires were used to make subjects score subjectively on the usability, trust, workload and affective dimensions using a Likert scale. Usability was measured using the After-Scenario Scale (ASQ) [34], which combined three dimensions of ease of task completion, the time required to complete tasks, and satisfaction with support information to produce an evaluation. The trust scores in the study were based on the model of trust in vehicle automation proposed by Muir [35], using a post-task trust scale with a comprehensive analysis of three trust dimensions: predictability, dependability, and faith. It was found that human reliance on automation was influenced by workload [36]. We used the Driving Activity Load Index (DALI) scale [37], which is a scale that concerns multi-channel information and includes the effort of attention, visual demand, auditory demand, temporal demand, interference, and situational stress. The DALI scale is more fit for dynamic driving conditions. The SAM scale [38] designed by Bradley et al. was used to measure the emotional state of the person in terms of pleasure, arousal, and dominance. In the comparison of the analysis of the result between specific experimental groups, priority was given to safety, followed by usability, trust, and workload ratings. Emotional ratings were also taken into account as a secondary evaluation.

### 4.4. Apparatus and Materials

**Figure 4:**
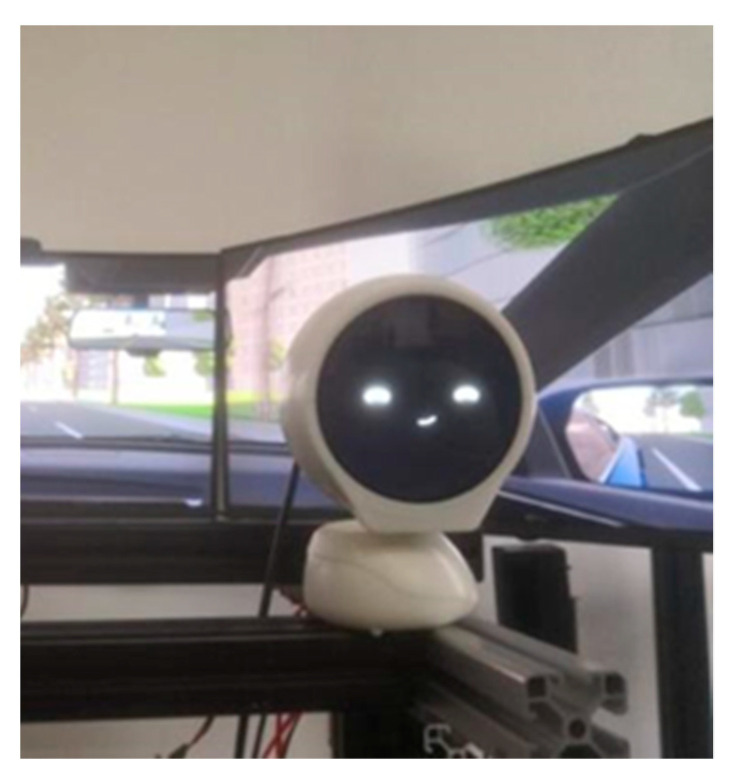
Robot for experiment.

**Figure 5:**
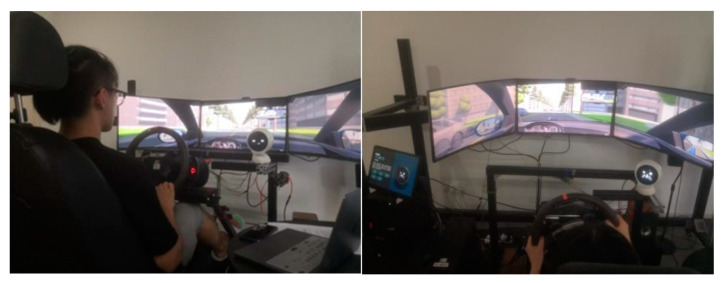
Experiment environment.

**Figure 6:**
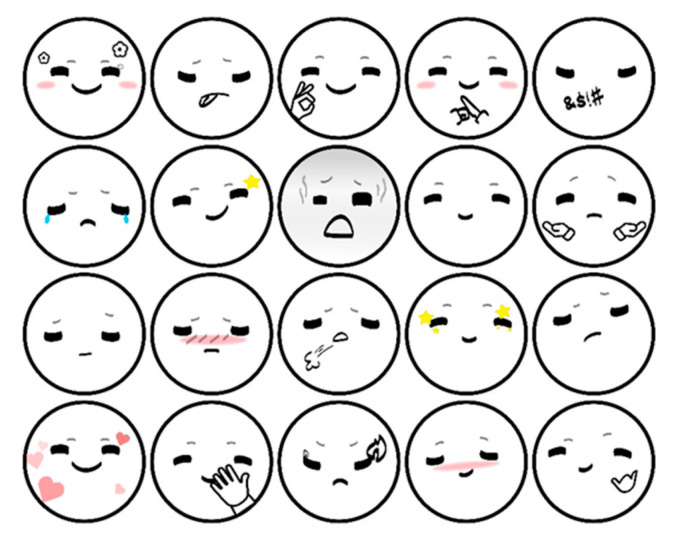
Expression design set used on robot’s face screen [39].

The experimental environment was built based on the driving simulator system independently developed by the Car Interaction Design Lab of Tongji University. The simulator was used as the main equipment of the experiment. The scene was developed using Unity software to simulate the real driving environment. The scene used in the experiment was a two-lane straight road with a large number of oncoming cars in the opposite lane. The simulator was equipped with monitoring equipment that automatically collected vehicle data during each simulation. The robot was fixed in a suitable position (see Figure 4). The location of the robot is determined by three factors: first, previous work by Williams [24] and others showed that in-vehicle robots were fixed in a position above the center screen. Second, we conducted a real vehicle study on nomi, the in-vehicle robot of Nio, measuring the relative position of the in-vehicle robot to the center of the steering wheel in three-dimensional space. Third, we placed the robot on our simulator and conducted a small test on the pre-fixed robot to adjust its position to make it closer to the real driving environment (see Figure 5). The vehicle robot had three degrees of freedom and was controlled by servos that can raise and lower its head and rotate toward the driver. The robot’s face screen displayed features and colorful auxiliary graphics to express expressions, which was in-depth explored and designed in our previous research [39]. The expression design was shown in Figure 6, in which the color and brush strokes were adjusted due to the confidentiality of company cooperation. The in-vehicle robot was accompanied by an interactive simulation program to control the robot’s movements and expressions. The interactive simulation program can record the robot’s behavioral data. Since the program requires the control of the experimenter, factors such as the experimenter’s reaction time may pose a threat to the validity of the experiment. The subject’s basic information form and scale were used to collect subjective data. Two cameras were used to record the visual behavior of the subjects. After the videos were recorded, the user’s saccades of the robot were manually checked frame by frame. The number of saccades time and fixation time were recorded with a minimum frequency unit of 0.042 s (1/24 s per frame). Similar simulator construction and data acquisition methods also appeared in other contemporary studies [40].

### 4.5. Procedure

The experimental consent forms were signed before the experiment, and the subject was registered for basic information including driving frequency as well as in-vehicle robot understanding and experience. Before conducting the experiment, the subject was introduced with the purpose of the experiment and the main tasks. The subject started with a driving in the simulator for about five minutes to learn and became familiar with the simulator. Then, the subject interacted with the in-vehicle robot in a simulation. After the subject was familiar with the simulator and the in-vehicle robot, the experiment started, and the experimenter began data recordings.

The tester described the driving task requirements and subtask requirements to the subject. After the subject confirmed, the tester issued the command to start the task. Subjects completed the telephone task first and the speeding task second. In each task, subjects were assigned to one of the groups. In the telephone task, the subject was asked to keep driving in the left lane at a speed of 30 km/h. After the subject kept driving stable, the experimenter simulated a phone call from the subject’s friend Wang. Then, the robot took the initiative to alert and ask the person whether to answer it. In the speeding task, the subject was asked to drive in the left lane at a speed of 30 km/h and then accelerated to 70 km/h. Once the speed reached 70 km/h, the robot took the initiative to remind the speed limit. After each task was completed, the subject completed the subjective questionnaires and scales. Then, the tester conducted the interview regarding the task. Each task lasted approximately five to ten minutes. After tasks were completed, subjects were interviewed by the tester regarding the in-vehicle robot generally.

## 5. Results

T-tests were conducted in the telephone task and one-way analysis of variance (ANOVA) with post hoc pairwise comparison was conducted in the speeding task. A summary of the experimental results in separate tasks can be seen in the following sections.

### 5.1. Telephone Task Results

**Figure 7:**
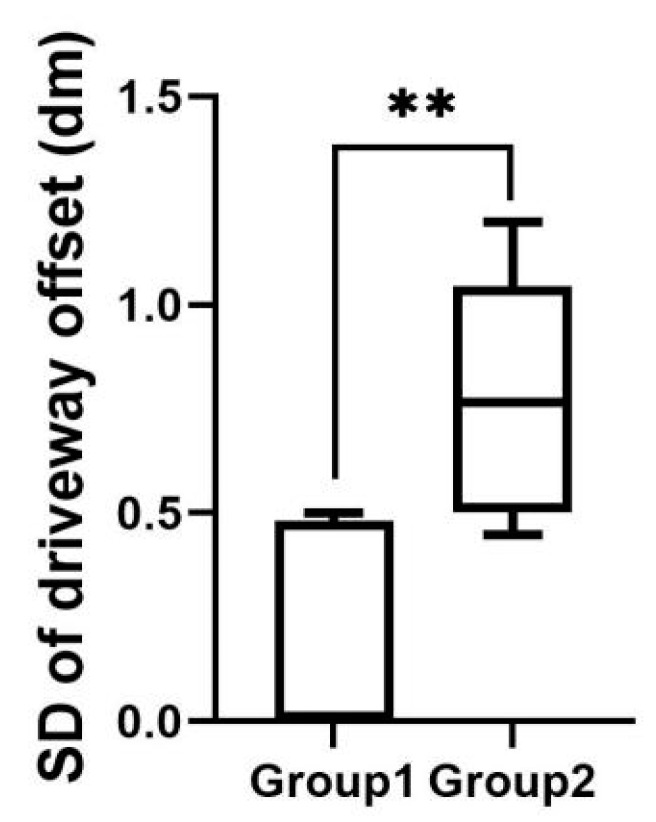
Standard deviation of driveway offset in telephone task. ** indicates significant difference (*p* < 0.01).

As shown in Figure 7, the mean value of the standard deviation of driveway offset for experimental group 1 was 0.18, and the mean value of the standard deviation of driveway offset for experimental group 2 was 0.77. There was a significant difference between the two experimental groups (p=0.00<0.01).

**Figure 8:**
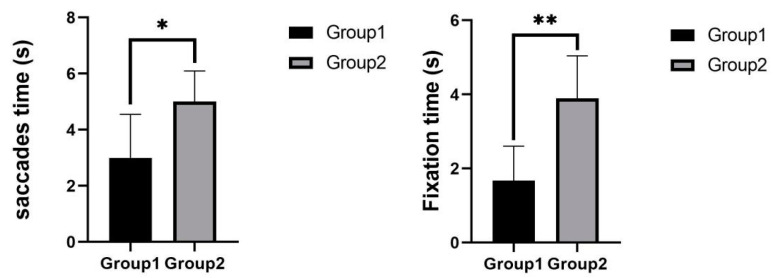
Saccades time and fixation time in telephone task. * indicates significant difference (*p* < 0.05). ** indicates significant difference (*p* < 0.01).

As shown in Figure 8, the mean saccades time was 3 s for experimental group 1 and 5 s for experimental group 2. There was a significant difference between the two experimental groups (p=0.02<0.05). The mean fixation time was 1.671 s for experimental group 1 and 3.893 s for experimental group 2. There was a highly significant difference between the two experimental groups (p=0.00<0.01). Subjects in experimental group 1 had significantly fewer saccades time and fixation time than subjects in experimental group 2.

**Figure 9:**
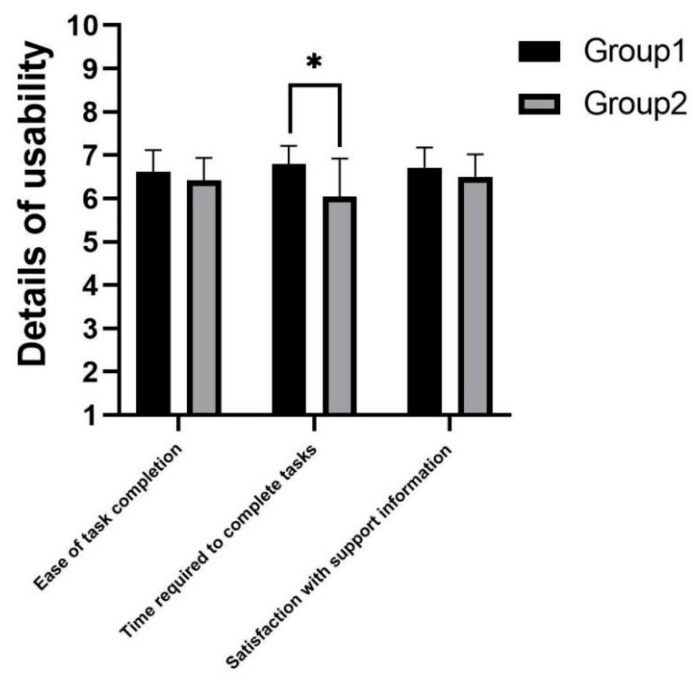
Usability in telephone task. * indicates significant difference (*p* < 0.05).

As shown in Figure 9, it can be concluded from the usability scores that the experimental group 2 subjects rated the usability of the robot lower (p=0.049). In terms of details, the time required to complete tasks was significantly different between experimental groups (p=0.01), while ease of task completion (p=0.374) and satisfaction with support information (p=0.262) was not significantly different.

**Figure 10:**
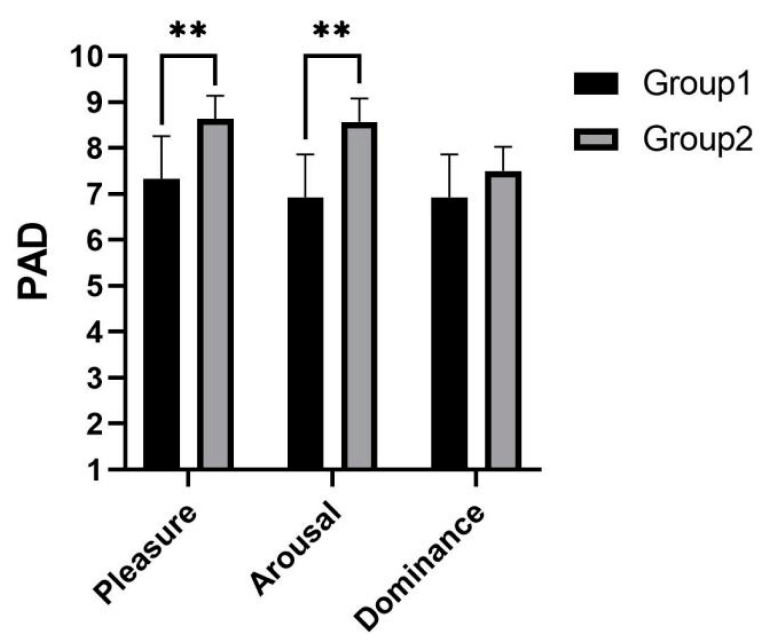
Emotion score of telephone task. ** indicates significant difference (*p* < 0.01).

As shown in Figure 10, it was concluded from the PAD scores that experimental group 2 enabled subjects to obtain more positive emotions. The subjects were more pleasant (p=0.001) and aroused (p=0.001) than experimental group 1. There was no significant difference in dominance (p=0.202).

**Figure 11:**
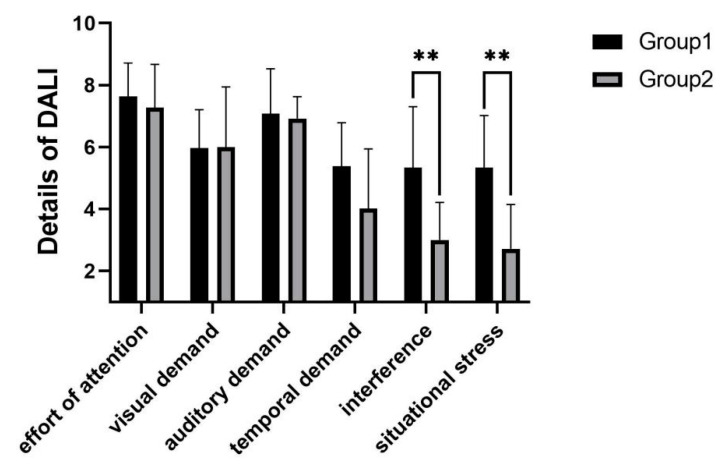
Workload in telephone task. ** indicates significant difference (*p* < 0.01).

As shown in Figure 11, it can be concluded from the workload scores that in experimental group 2, subjects had a lower workload than in experimental group 1 (p=0.002). Judging from the DALI detail scores, the main reasons were lower interference (p=0.003) and lower situational stress (p=0.002).

### 5.2. Speeding Task Results

**Figure 12:**
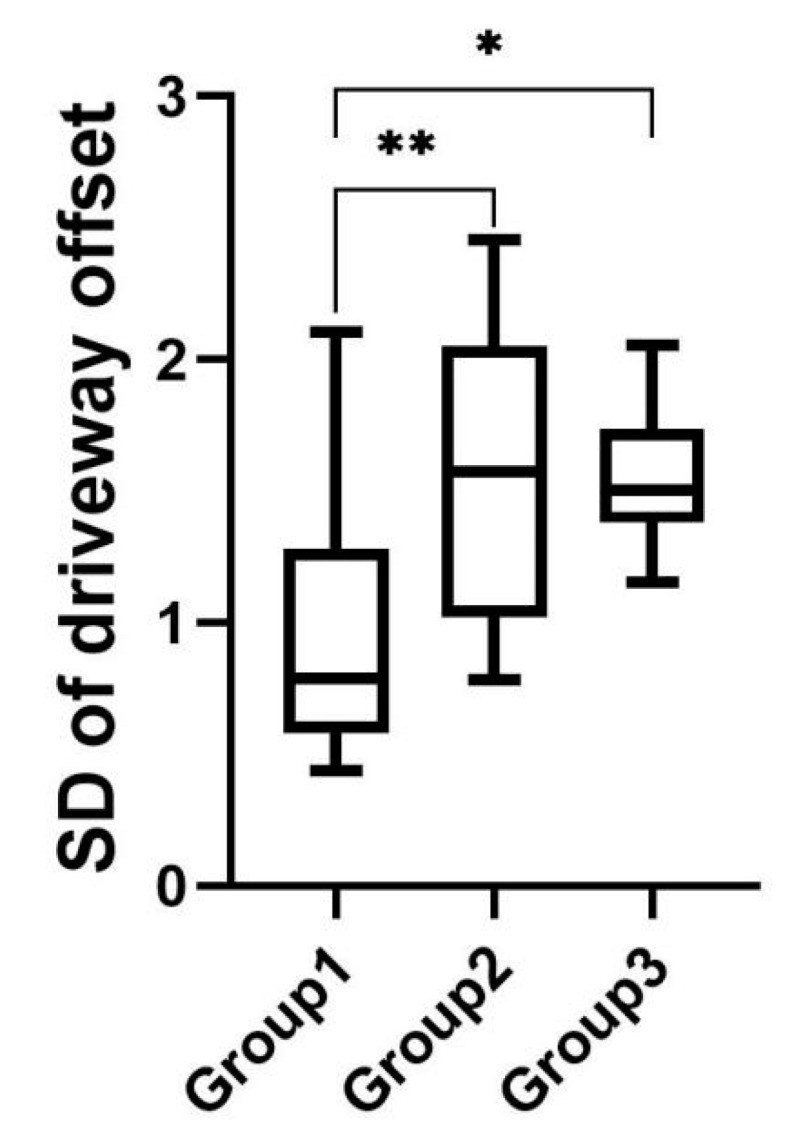
Standard deviation of driveway offset in speeding task. * indicates significant difference (*p* < 0.05). ** indicates significant difference (*p* < 0.01).

As shown in Figure 12, the mean value of the standard deviation of driveway offset was 0.95 for experimental group 1, 1.54 for experimental group 2, and 1.55 for experimental group 3. The results of the one-way ANOVA performed on the data from the three groups indicated that there was a significant difference in the standard deviation of driveway offset among the three experimental groups (F(2,32)=6.906, p=0.00<0.01); post hoc tests revealed that the standard deviation of driveway offset was significantly lower in experimental group 1 (M=0.97, SD=0.51) than in experimental group 2 (M=1.60, SD=0.58) and experimental group 3 (M=1.55, SD=0.28), with no significant differences between experimental group 2 and group 3.

**Figure 13:**
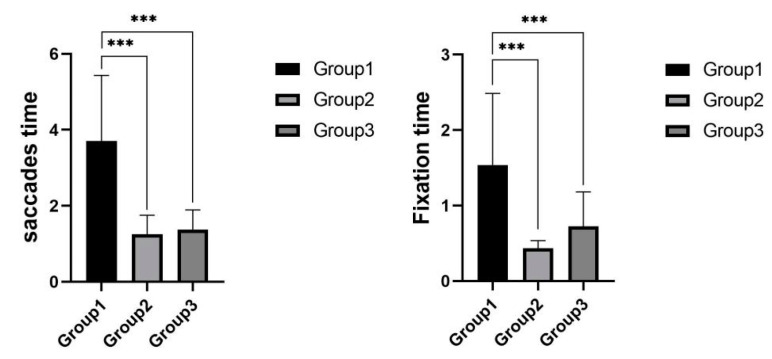
Saccades time and fixation time in speeding task. *** indicates significant difference (*p* < 0.001).

As shown in Figure 13, the mean saccades time was 3.7 s for experimental group 1, 1.3 s for experimental group 2, and 1.4 s for experimental group 3. One-way ANOVA results of the data from the three groups showed that there was a significant difference in the saccades time in the three experimental groups, (F(2,18)=9.479, p=0.00<0.01); post hoc tests revealed that the saccades time in experimental group 1 (M=3.67, SD=1.73) was significantly higher than that in experimental group 2 (M=1.25,SD=0.50) and experimental group 3 (M=1.38,SD=0.52), with no significant difference between experimental group 2 and experimental group 3. The mean fixation time of experimental group 1 was 2.389 s, the mean fixation time of experimental group 2 was 0.540 s, and the mean fixation time of experimental group 3 was 0.834 s. A one-way ANOVA of the data from the three groups showed that there was a significant difference in the fixation time of the three experimental groups, (F(2,20)=7.245, p=0.00<0.01); post hoc tests revealed that the fixation time of experimental group 1 (M=2.39,SD=1.41) was significantly higher than those of experimental group 2 (M=0.54,SD=0.30) and experimental group 3 (M=0.83,SD=0.50), and there was no significant difference between experimental group 2 and experimental group 3.

The results of the behavior data showed that the subjects in experimental group 1 were significantly higher than those in experimental group 2 and experimental group 3 in terms of saccades time and fixation time.

**Figure 14:**
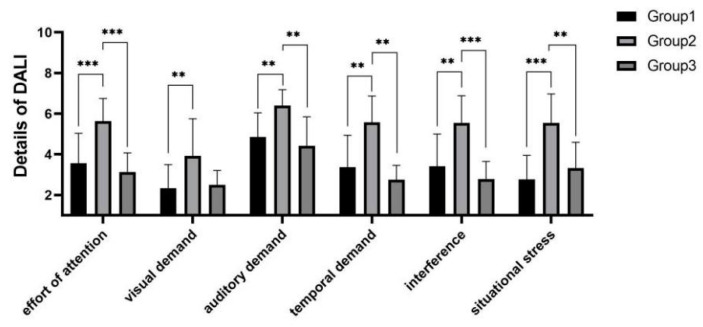
Workload in speeding task. ** indicates significant difference (*p* < 0.01). *** indicates significant difference (*p* < 0.001).

As shown in Figure 14, significant differences were shown between experimental groups in terms of workload score means (F(2,55)=10.408, p=0.000<0.001) and also in terms of each detailed dimension. Post hoc test analysis yielded that the workload of experimental group 2 was significantly higher than those of experimental group 1 (p=0.000<0.001) and experimental group 3 (p=0.002<0.01), while experimental group 1 and experimental group 3 did not show significant differences in both workload score means and workload score details.

**Figure 15:**
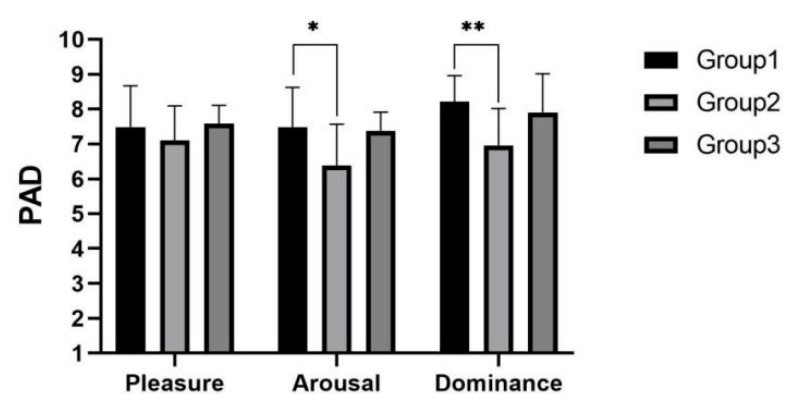
Emotion score in speeding task. * indicates significant difference (*p* < 0.05). ** indicates significant difference (*p* < 0.01).

As shown in Figure 15, it can be concluded from the PAD scores that there were significant differences among three experimental groups in the arousal (F(2,38)=3.430, p=0.043<0.05) and dominance (F(2,42)=5.945, p=0.005<0.01) dimensions. Post hoc test analysis yielded significantly higher arousal (p=0.036<0.05) and significantly higher dominance (p=0.004<0.01) in experimental group 1 than in experimental group 2.

## 6. Discussion

In the telephone task, the design with SAT1 perception level, the voice channel information (hereafter referred to as the ringing group) in group 2 enabled subjects to obtain more positive emotions and reduced the workload of the subjects. This was probably because the added information enhanced the anthropomorphism of the robot. However, subjects had significantly worse horizontal control of the vehicle, and the saccades time and fixation time increased significantly. This suggests that the subjects were overly distracted by the robot and had reduced concentration on the driving task, which may cause danger. As for the subjective data, subjects rated the usability of the ringing group lower, and significant differences were found mainly in the aspect of time required to complete tasks. It can be obtained that task disfluency due to lengthy speech triggered subjects’ lower usability ratings of the robot. In addition, the increased information at the expense of time spent did not improve usability ratings in terms of ease of task completion and satisfaction of support information. Overall, the existence of SAT1 perception level provide higher transparency. However, it does not suggest better cooperation between human and robot.

In the speeding task, the design with SAT2 comprehension level presenting voice and visual channel information as well as SAT3 projection level showing voice channel information had better information transparency. In terms of safety, group 1 had the best data performance in terms of vehicle horizontal control. Adding the SAT3 projection level visual channel information or SAT1 level voice channel information would make the driver’s horizontal control of the vehicle worse. From the subjective data, having SAT3 level visual channel information resulted in significantly higher workload and lower arousal and dominance. The speed limit expression at the projection level caused subjects’ greater workload and did not help in driving, indicating that it was an unnecessary distraction for the driver. The presence or absence of SAT1 level with voice channel information (‘oops’) did not significantly affect workload. Therefore, in the speeding task, the design of the in-vehicle robot can omit the SAT1 stage information, which is not very helpful for the task, and the complicated SAT3 level visual information can be similarly excluded to increase safety and reduce the workload.

In summary, we analyzed the appropriate amount of transparency information for in-vehicle robots in a proactive interaction scenario. The experimental groups with SAT1 performed poorly in both scenarios. In order to improve the effectiveness of message delivery and help drivers concentrate, designers should carefully consider increasing voice channel information at the SAT1 perception level. The design that performs better in telephone tasks has SAT2 comprehension and SAT3 projection levels of both voice and visual channel information, which is different from the better design for speeding tasks. In the speeding task, the SAT3 projection level and visual information make the workload of subjects significantly higher, and the driving safety is compromised. Analyzing the task characteristics, we can conclude that the telephone task belongs to function, while the speeding task belongs to critical scenarios. The driver’s concentration level on driving tasks in the two scenarios is different; therefore, the driver needs different robot transparency.

The advantage of higher transparency for the in-vehicle robot is that it can help drivers judge the situation, reduce drivers’ need to reprocess the information provided by the robot, and increase the drivers’ decision-making confidence. However, higher transparency also has its own disadvantage. In the case of insufficient attention and mental resources, too much information is not only not fully received by the human but also affects the driver’s driving performance, especially when implicit information such as robot expressions that require extra mental resources from the driver appears. These results suggest that the ‘highly transparent’ assumption made by previous work needs careful consideration. In critical scenarios, it is recommended to design more direct voice information without adding extra workload. In non-critical driving scenarios, designers can consider giving robots a more transparent response to drivers while maintaining task fluency.

The experiments were based on transparency design assumptions, and they yielded preliminary results for the exploration of suitable transparency. We are able to draw enlightening conclusions from different measurements, and the purposes of the experiments are essentially achieved. However, due to the experimental limitations, it was not possible to exhaust the possible design assumptions, and there may be more appropriate transparency designs for specific scenarios.

The relationship between saccades time and driving safety was also noted in both scenarios. In the non-critical scenario, an increase in saccades time and fixation time on the robot undermines driving safety. In the critical scenario, on the contrary, an increase in saccades time and fixation time of the robot improves driving control. The reason for this result derives from the difference in the type of tasks that the driver and the in-vehicle robot achieve together in the two scenarios. Taking workload into comprehensive analysis, in the case of joint driving tasks such as the speeding task, the robot’s information alerts can share the workload for the driver and obtain better driving performance. However, when the robot is completing non-driving tasks such as picking up the phone, the increase in saccades time and fixation time indicates that the driver is more involved in non-driving tasks and does not concentrate on driving tasks, so the driver tends to have poor control of the vehicle. Further analysis revealed a tendency for the reduced self-reported workload when drivers were more involved in non-driving tasks. This may be due to the higher workload of the driving task compared to the non-driving task, which is also able to corroborate with the multi-resource prediction of dual-task interference in the multi-resource theory (MRT) proposed by Wickens [41].

## 7. Conclusions and Future Work

In this paper, we migrate SAT theory into an in-vehicle robot design method and conduct a proactive interaction design of in-vehicle robots based on transparency design assumptions. The results of the experiment show that the driver’s driving control, behavior, and self-reporting results produce differences under different in-vehicle robot transparency. Overall, anthropomorphic information of perception level, though bringing a better emotional experience to the driver, can cause poorer driving performance. In the critical scenario, because of the need to ensure timely and effective information, the driver can accept lower information transparency of the in-vehicle robot than in the non-critical scenario, retaining only key information. The conclusions can be distilled into design guidance: in the in-vehicle scenario, information transparency design assumptions can be made for both voice and visual channels based on SAT theory to help designers arrive at interaction solutions with more appropriate transparency. The guidance can also give a methodology to solve the transparency challenge that lies in future research of human-centered shared control [42]. Following the guidance, this paper verifies that in the proactive interaction scenario of in-vehicle robots, non-critical scenarios require comprehension and projection level information, while the projection level information can be reduced in critical scenarios.

At this stage, the multi-channel interaction design is not separated from transparency. The multi-channel content is only used as an independent variable of transparency change to serve the transparency design. In terms of future work, we will go further to establish the design model integrating multimodal and transparency, concerning input and output modalities [43], and then add subsequent experiments to validate it. A study shows that EEG-based measurements can be a powerful tool for studying driver behavior [44]. It is also able to measure the effect of multimodal information on cognition very accurately [45]. Psychological precision measurement instruments such as EEG, ECG, or eye movements have not been included in the scope of measurement methods in this paper, and further work needs to consider bringing such measurement tools methods into the experiment.

## Figures and Tables

**Table 1:** Transparency design assumptions in proactive interaction condition of the in-vehicle robot.

	Voice	Visual
SAT1	?	/
SAT2	√	√
SAT3	√	?

The symbol “√” means the information is needed; the symbol “?” means it cannot be determined whether the information is needed and needs proving; the symbol “/” means the information is not needed.

## Data Availability

Data sharing is not applicable.
